# Bibliographical

**Published:** 1883-03

**Authors:** 


### Bibliographical.

*Spare Hours.* JOHN BROWN, M.D. Boston: Houghton, Mifflin & Co. 1883. 3 vols., pp. 375, 450 and 425, respectively.

Under the title "Spare Hours,"—a phrase that has an idealistic sound to most practitioners—Dr. Brown has written a number of stories, reminiscences, memoirs and critiques that for variety, interest, quaint phraseology and deep, keen and loving humanity both towards man and the brute creation, can not be matched in our literature.

The medical world should be proud of one who, while steadily and thoroughly practising his chosen and domineering profession, can still fill no mean place in a still wider field—general literature.

We doctors take an interest in whatever is done—if done well—by one of us outside the pale of the regulation practice. Witness, for instance, how Dr. Haden, in his lectures on "Etching," is chronicled in the American press. And did not Sir Thomas Watson, Sir Henry Thompson, Dr. Richardson and our own Hayes and Holmes labor in other fields than that of medicine?

Recently books have been published telling doctors how to dress, talk, act, how to have their offices furnished, and advising them to form, if any new acquaintances, those of their own "business;" that is the term, we think, adopted in such books.

Breadth in any calling is obtained only by those minds who associate with books and men who do not eternally remind them of *the shop,* who have other interests, and who push them with as true and devoted energy as most physicians do theirs.

In the quaintly serious "sermons" of our author anent "The Doctor," we find him telling us to "trust, obey, speak the truth to and reward him. You must reward him by giving him your money, your gratitude, by doing his bidding, and, lastly, by speaking well of him, giving him a good name, recommending him to others."

The "sermon" to the doctor upon his duties to you, *i.e.*, the laity, is too good to be quoted piecemeal. In one of his lay sermons to working people, he says: "It is the doctor's duty to keep *his time and temper with you.* Any man or woman who knows how longed for a doctor's visit is, and counts on it to a minute, knows how wrong, how painful, how angering it is for the doctor not to keep his time. Many things may occur, for his urgent cases are often sudden, to put him out of his reckoning; but it is wonderful what method, and real consideration, and a strong will can do in this way. I never found Dr. Abercrombie a minute after or before his time (both are bad, though one is the worser,) and yet if I wanted him in a hurry, and stopped his carriage in the street, he would always go with me at once; he had the knack and the principle of being true to his times, for it is often a matter of truth." The books are full of honesty of mind, not dwelling in those methods by which you can gain customers like an importunate and impecunious tradesman.

For a rest after study, doubly pleasing from style and brevity [illegible] Spare Hours of the versatile Scotsman are delightful; and a physician library would be incomplete indeed, had he never so few spare hours, were "Dr. Broon" not to find a place along his shelves.

*Functions and Disorders of the Reproductive Organs.* William Acton, M.R.C.S., Sixth Edition. Philadelphia: P. Blakiston, Son & Co. 1883.

On closing this book we are struck with the thought, that if those who needed its sound and convincing advice, could or would read it, much good would result, for it is an outlay in the right and proper form of all that is perverted in quack productions, hinted at in the columns of so many of our dailies, and thrust in the hands of passing youths in the form of advertisements by dirty tramps, hired by the quacks infesting our cities.

Dr. Acton reasons, pleads with, and gives the onanist, debauchee, and all who give sexual passion unbridled sway, the very best advice. He quotes Juvenal, Shakspere, Horace, most of the dictionaries, and a vast number of physiologies,—but where is his audience?

Six editions must mean something, certainly; but unless men outside of the profession buy and read it, we must either suppose the doctors all want the book, or else that they buy it and give it to the "erring ones." The latter we do not think likely. And as every well-read, common-sense physician knows the anatomy and physiology of the genital organs, and the derangement—both mental and physical—induced by any disease or disturbance in function of "this delicately arranged apparatus," he cannot learn very much from Doctor Acton.

Several "spicy" cases are given, and, for the salacious, might result in morbid thought, or what is called here, "mental masturbation," if other than the physician opened its pages.

At any rate, you would not leave the book upon the library table, since it contains a history of nearly every vile practice that man or woman resorts to.

Still, it is full of honesty and common sense from one end to the other; it abounds in quotations that show how sincere the author feels in his work, and how deeply he has read in all the literature that bears, in any way, upon his subject.

One thing in his book is wrong, to name none other, and that is where he ascribes to ladies and gentlemen who dance the so-called "round dances" together erotic feelings, stating that chronic uterine disease results therefrom, in the "fashionable women of to-day."

Rather to fashion, late hours and close rooms would we ascribe the uterine disorders, than to the few minutes of "a dance." None but a few animals, at best, could be so excited, and those would not *dance* for the purpose, be sure. In this and a few other matters our author carries it too far. His book is on an important subject; but a subject that does not take in quite *all* the domain of medicine and society ills, as he would have us believe.

*A Practical Treatise on Operative Dentistry.* J. Taft, M.D., D.D.S. Fourth edition, revised. Philadelphia: P. Blakiston, Son & Co. 1882. Price, cloth, $4.25; sheep, $5.

This work is already too well known in the profession to need any extended review at our hands. In all the literature of Dentistry there is nothing which has met with greater favor, or been more extensively adopted as a text book. The very fact that a fourth edition was demanded is quite sufficient proof of the high estimation in which it is held by dentists. Its author is so well known, both as a teacher and writer, that his statements are usually accepted without controversy by most practitioners. Yet since the first edition was published our knowledge of dental tissues has been so continually extended, and dental practice has been so modified, that a standard work like this needs constant revision. There is more than one statement in the book which, once in harmony with the prevalent pathological views of the day, is now, in the estimation of many intelligent men, antiquated and obsolete. But, while discussion is yet active over these disputed questions, and until they are authoritatively settled, the author has, in our opinion, done well to stand by his first utterances. It is not at all improbable that, as the result of investigation now being actively pursued, dental practice will in the near future need yet further material modification, and the fifth edition may be profitably re-written. It is not wise in a standard text book like this, to incorporate as a part of an established practice methods, materials and remedies, the merits of which are now in dispute. Until the time shall come when the present transition state of dental practice shall have passed, and the whole profession shall be better agreed upon certain controverted questions, the day for the complete revision of Taft's Operative Dentistry has not arrived. When that time shall come the limits of a volume like this will not contain all that should be incorporated in it, and a practically new work will be the result. Until that period, the book under notice will probably remain what it has been in the past, the best work upon operative dentistry extant.

The publishers have done their whole duty by the author ; the type is exceptionally clear, the cuts are well executed, and the whole volume presents a handsome appearance.

*Chance's Dental Itemizer ; embracing an entirely new system for quickly and accurately recording all dental operations.* Devised and arranged by Geo. H. Chance, D.D.S., Portland, Oregon.

This is the most concise and succinct of the many methods for the registering of dental operations. It comprises a system of arbitrary characters, however, which must be first committed to memory and made entirely familiar to the eye, after which the proper record is easily kept, and forms a very complete history of the work done for each patient.

*Scrofula and its Gland Diseases. An introduction to the general*

*pathology of Scrofula.* Frederick Treves, F.R.C.S., Eng. Philadelphia: H. C. Lea's Son & Co. 1883. Pp. 77, Price, ten cents.

This cheap little pamphlet has in condensed form, most of the modern views succinctly formulated. There is no index and but a sparse survey in the table of contents. We look in vain for Koch's name or anything concerning his experiments.

Pamphlets Received.—*Annual Reports of the Sanitary Protective Association of Newport, R. I. Transactions of the N. H. Medical Society,* at its Ninety-second Annual Session, held at Concord, N. H., June, 1882. *Fifteenth Annual Report of the New York Orthopœdic Dispensary and Hospital* (for children with spine and hip diseases and other deformities), for the year ending September 30, 1882.

*The Proceedings of the Medical Society of the County of Kings* Vol. 7. No. 12. The major part of this number is devoted to an aricle by Dr. N. B. Size, entitled "Resorcine—Its History and Antiseptic Value."

We cull the following from p. 273 of the "Proceedings :"

Mr. A. sold a colt, as a gelding, to Mr. B., which colt had had but one testicle removed, the other remaining within the cavity of the abdomen. The veterinary surgeon who had castrated the animal was sworn, and, on his cross-examination, stated the following interesting features in the anatomy of the horse :

*Attorney*—What are varicose veins and where are they found?

*Witness*—I don't know, but I can tell where the bellicose veins are.

*Atty.*—Where are they?

*Wit.*—Close to the belly.

*Atty.*—Where is the scrotum?

*Wit.*—I am not quite certain, but I think that it is the film that covers the teeth during infancy.

*Atty.*—Have you ever made any examinations in the abdominal region?

*Wit.*—No; all of my examinations have been made in Broome County.

The annual meeting of the New York Medical Mission was held at the Broadway Tabernacle on Tuesday evening, February 20, at 8 o'clock. Prof. A. C. Post, M.D., LL.D., presided. Bishop Harris, Dr. Brachet, of China, and Rev. Drs. Taylor, Vincent and Sabine delivered addresses.

